# Expertise with unfamiliar objects is flexible to changes in task but not changes in class

**DOI:** 10.1371/journal.pone.0178403

**Published:** 2017-06-02

**Authors:** Rachel A. Searston, Jason M. Tangen

**Affiliations:** 1 Melbourne Centre for the Study of Higher Education, Melbourne Graduate School of Education, The University of Melbourne, Melbourne, Australia; 2 School of Psychology, The University of Queensland, Brisbane, Australia; University of Melbourne, AUSTRALIA

## Abstract

Perceptual expertise is notoriously specific and bound by familiarity; generalizing to novel or unfamiliar images, objects, identities, and categories often comes at some cost to performance. In forensic and security settings, however, examiners are faced with the task of discriminating unfamiliar images of unfamiliar objects within their general domain of expertise (e.g., fingerprints, faces, or firearms). The job of a fingerprint expert, for instance, is to decide whether two unfamiliar fingerprint images were left by the same unfamiliar finger (e.g., Smith’s left thumb), or two different unfamiliar fingers (e.g., Smith and Jones’s left thumb). Little is known about the limits of this kind of perceptual expertise. Here, we examine fingerprint experts’ and novices’ ability to distinguish fingerprints compared to inverted faces in two different tasks. Inverted face images serve as an ideal comparison because they vary naturally between and within identities, as do fingerprints, and people tend to be less accurate or more novice-like at distinguishing faces when they are presented in an inverted or *unfamiliar* orientation. In Experiment 1, fingerprint experts outperformed novices in locating categorical fingerprint outliers (i.e., a loop pattern in an array of whorls), but not inverted face outliers (i.e., an inverted male face in an array of inverted female faces). In Experiment 2, fingerprint experts were more accurate than novices at discriminating matching and mismatching fingerprints that were presented very briefly, but not so for inverted faces. Our data show that perceptual expertise with fingerprints can be flexible to changing task demands, but there can also be abrupt limits: fingerprint expertise did not generalize to an unfamiliar class of stimuli. We interpret these findings as evidence that perceptual expertise with unfamiliar objects is highly constrained by one’s experience.

## Introduction

Identifying an object in a new context, view, and orientation is one of the most formidable visual tasks that we face. There is often a great deal of *within*-object variation (e.g., flowers wilt, fruit rots, people age, and appearances change with position, lighting, and scale) and *between*-object similarity (e.g., dandelions are often confused with hawkweed, peaches are essentially fuzzy nectarines, and siblings tend to look alike [1], [2]). We rely on our memories for prior instances to help resolve a lot of this complexity [3], [1]. As a result, our ability to distinguish objects tends to be highly constrained by our experience [4], [5], [6]. A well documented illustration of this problem is the cost we incur when attempting to recognize familiar faces and objects when they are presented in an unfamiliar, inverted orientation [7], [8], [9], [10], [11]. Recognition is made even more difficult when the object itself is novel or less familiar to us. Most notably, unfamiliar faces are difficult to recognize across changes in view, compared to familiar faces [12], [13], [14], [15]. The highly specific nature of perceptual expertise has been demonstrated time and again, and in domains as diverse as car recognition [4],Tetris [16] and chess [17].

Despite the difficulty in identifying novel or unfamiliar instances, objects, identities, and categories, there are experts in forensic and security settings who learn to distinguish between unfamiliar images, such as an unknown fingerprint or face, without any opportunity to accumulate specific experiences at the source or object level [18], [19]. Forensic examiners spend their days visually comparing images they have never seen before, side-by-side, to determine whether, for example, a pair of fingerprints or photographs originated from the same object (Smith’s left thumb or Smith) or different objects (Smith’s left thumb and Jones’s right middle finger or Smith and Jones). For the fingerprint or face expert, each case is a new instance of an *unfamiliar* finger or identity and, aside from their vast general experience with the broader domain of ‘fingerprints’ or ‘faces’, the only object-specific instances at their disposal are in front of them (rather than in memory). In other words, these experts have no prior experience to draw on about how the objects they are distinguishing (e.g., Smith’s left thumb from Jones’s right middle finger or Smith’s photograph from Jones’s photograph) typically look and vary across images (e.g., multiple images of Smith’s left thumb or face).

In these real-world perceptual domains, a genuine expert is considered to have demonstrable abilities above and beyond less experienced observers, who in many cases are members of the jury [20]. We rely on these unfamiliar face and object matching experts to detect passport fraud or to identify individuals who were at the scene of a crime, but what is the basis for their expertise when each object they encounter is unfamiliar to them? Is their expertise bound by the same specificity effects seen in other domains of expertise where the objects are familiar?

### A general perceptual skill

Findings from the unfamiliar face matching literature provide evidence that expertise with unfamiliar objects may rely on a superior general perceptual skills. One investigation found no significant relationship between how well undergraduates match familiar faces (e.g., photographs of a friend, famous person, or family member), and how well they match faces of people they’ve never encountered before [21]. When the faces were flipped upside-down, however, their matching performance with the inverted (familiar and unfamiliar) faces and upright unfamiliar faces was strongly correlated. The authors interpret this result as evidence of a *qualitative* shift in processing as people become familiar with a particular identity. In other words, inverted and unfamiliar faces both appear to be processed like a novel stimulus class, relying on a different and more general perceptual mechanism than familiar faces. Consistent with this viewpoint, expert unfamiliar face matchers in passport and security settings are also less impaired by inversion than undergraduates (although this same effect was not found when comparing expert examiners to professional controls; [19], see also [22]).

These findings in the unfamiliar face matching literature are counterintuitive because in other documented examples of the inversion effect, performance with inverted images of a given category tends to decline with more experience and expertise (with the upright category [9]). This effect is commonly cited as evidence of non-analytic, holistic or configural processing, whereby the images are recognized as a whole, by gleaning the relational or covariant visual structure among constitute features, rather than part-by-part or by a general perceptual process [23]. The opposite appears to be the case, however, with unfamiliar face matching examiners, suggesting they may rely less on configural processing than in domains where the objects or categories are familiar. If this is true, these unfamiliar face matching studies provide some indication that expertise with unfamiliar objects (or identities) may rely on a more *general* perceptual skill—one that is more resistant to changes in orientation, and stimulus class.

### An instance-based skill

An alternative perspective is that unfamiliar face and object matching experts are relying on their memory for how instances of fingerprints or faces generally tend to vary (across fingers, individuals, and contexts) to help resolve novel cases. A memory retrieval process is characteristically fast, intuitive, and automatic, but limited by stored information [3], [24]. This perspective is consistent with accounts of perceptual expertise that suggest recognition of faces and objects relies on a similar cognitive process and that recognition of novel stimuli from an expert class is based on our experience with that general class [25], [26], [27], (for an opposing interpretation, see [28], [29]). For instance, several characteristics of face processing—such as inversion and misalignment effects—are observed, albeit to a lesser degree, in other object domains where people have developed expertise [7], [8], [9], [10].

Despite the paucity of within-object exemplars at their disposal (e.g., multiple impressions of Smith’s left thumb, or multiple photographs of Jones’s face), expert examiners display some characteristic traits of memory or instance-based expertise. Fingerprint examiners are more accurate than novices at distinguishing briefly presented prints [30]. They also show a delay in the N170 EEG component when viewing inverted versus upright fingerprint fragments (a physiological measure previously used to detect configural processing [31]; and are less impaired when matching fingerprints in artificial noise or when fingerprints are spaced briefly in time than novices [31], [30]. Unfamiliar face matching experts also maintain their advantage under speeded conditions [19]. Unfamiliar face matching performance, in general, suffers when the images are inverted [21], and novice fingerprint matching decisions are influenced by similarity to prior cases [32]. Taken together, these studies indicate that unfamiliar face and fingerprint experts make use of non-analytic, holistic, or configural processing when discriminating unfamiliar faces and objects within their domain of expertise, and suggest some reliance on information stored in memory.

### Present study

In the present study, we contrast people’s reliance on a general or an instance-based perceptual skill by probing whether fingerprint experts outperform novices across changes in task, and classes of stimuli. In Experiment 1, fingerprint experts and novices locate unfamiliar fingerprint categorical outliers among arrays of 40 fingerprints as quickly and as accurately as possible (i.e., locating a loop pattern in an array of whorls, or vice versa). We further test whether fingerprint experts maintain a performance advantage when classifying unfamiliar inverted face outliers using the same task (i.e., locating an inverted male face in an array of inverted female faces, or vice versa). In Experiment 2, we use a speeded matching task to probe how fingerprint expertise facilitates the discrimination of unfamiliar prints versus unfamiliar inverted face identities.

Our choice of inverted faces as a control stimulus was motivated by evidence that people tend to perform worse when face images are presented in an unfamiliar, inverted orientation [33]. That is, inverted faces, unlike birds or cars or other natural domains, are like a novel stimulus class to most people. Importantly, while fingerprints and inverted faces both vary naturally, they are not likely to share many of the same diagnostic visual regularities. Face identity is also analogous to finger identity (i.e., a particular finger is at a similar level of analysis to a particular face), and fingerprint experts tend to have years of experience with matching prints, but have limited experience with inverted faces. Furthermore, most people have no experience with fingerprints, thereby allowing us to contrast examiners’ performance with genuine novice controls. If experts outperform novices in locating and discriminating inverted faces as well as prints, we have evidence of a more domain-general perceptual skill that is robust to changes in stimulus class. Experts outperforming novices with fingerprints but not inverted faces, on the other hand, would suggest a form of expertise that is constrained by their memory for similar prior instances.

## Experiment 1

The purpose of Experiment 1 is to gauge the generalizability of examiners’ expertise outside their usual image matching task, and across two very different classes of stimuli. Fingerprint experts and novices are asked to complete two novel visual search tasks. In one, their goal is to locate a loop fingerprint pattern in an array of 39 whorl patterns (or vice versa). In the second, their goal is to locate an inverted female face in an array of 39 inverted male faces (or vice versa). Visual search paradigms have been useful in understanding the attentional and temporal demands of various visual processes, from the discrimination of low-level perceptual dimensions such as orientation or color [34], to the detection and categorization of more complex scenes [35] and objects, including faces [36], [37], [38]. Here, we chose to use a search paradigm with a large, fixed set-size of 40 where one of two target types (i.e., loop or whorl; male or female) was always present. The set size allowed us to maximize the difficulty of the task, while maintaining an image size where pattern detail could still be seen. The target and distractor trial types were randomly intermixed, such that the distractors on one trial became the target on another. Detecting the switch between targets and distractors encourages some degree of analytic processing [39], and a general perceptual ability ought to be less affected by this inconsistency.

**Fig 1:**
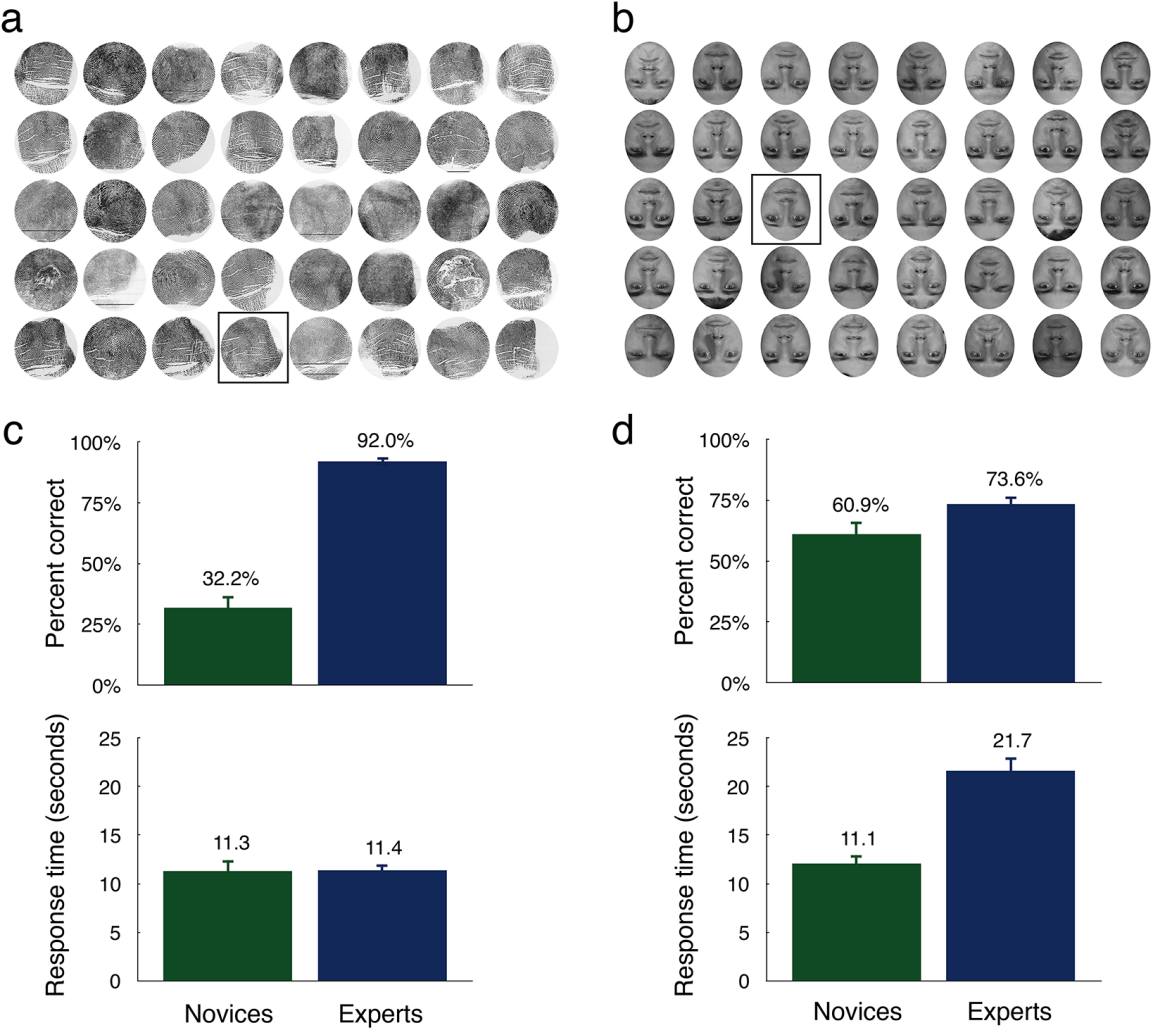
An example fingerprint array with a loop target (a), and an inverted face array with a female target (b). The mean percent correct and mean response time for novices (green) and experts (blue) on the fingerprint (c) and face (d) search tasks are depicted beneath each example array. Error bars represent the standard error of the mean.

**Fig 2:**
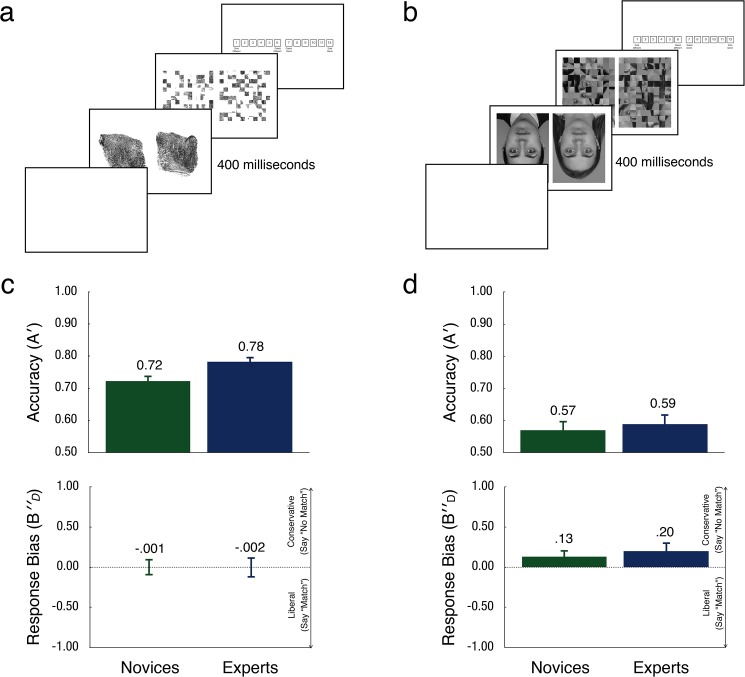
A representation of the fingerprint discrimination task sequence (a), and the inverted face discrimination task sequence (b). The mean accuracy (*A′*) or discrimination ability for novices (green) and experts (blue) on the fingerprint (c), and face (d) tasks are depicted beneath each sequence. Error bars show the standard error of the mean.

The research in this study was cleared in accordance with the ethical review processes of The University of Queensland and within the guidelines of the National Statement on Ethical Conduct in Human Research (Ethical clearance number: 2010000106). Participants provided verbal informed consent, and the consent procedure was approved by the ethics committee. Participation was recorded via an online participation platform, and the individuals in Figs 1 and 2 also provided informed consent to publish these case details.

### Participants and stimuli

#### Participants

We tested 16 practicing fingerprint experts (*Mean Age* = 39.25, *Standard Deviation =* 7.90; nine female) from four police organizations in Australia (Queensland, The Australian Federal, New South Wales, and Victoria Police) with an average of 12.5 years of experience. Sixteen undergraduates (*Mean Age* = 20.69, *Standard Deviation =* 6.91; 16 female) from The University of Queensland also participated for course credit as a novice comparison group.

#### Fingerprints

The fingerprints were 200 fully rolled impressions, 100 loops and 100 whorls, collected from different fingers of 30 individuals and sourced from the Forensic Informatics Biometric repository [18]. Loops and whorls are two common classifications used by fingerprint examiners to describe the general pattern of fingerprint impressions. The prints in the current set were classified by a qualified fingerprint expert in Australia with 15 years of experience. Each print was cropped to 180×180 pixels and we applied a circular mask, consistent with the face stimuli.

Forty arrays (20 containing a loop target singleton among whorl distractors and 20 containing a whorl target singleton among loop distractors) each consisting of forty fingerprints, were generated for each participant (see Fig 1A for an example of an array with a loop target singleton). A random sample of 20 whorls and 20 loops were set aside as targets for each participant, so no target was repeated in the experiment. For the distractors, 39 fingerprints were randomly sampled from the remaining pool of loops on the whorl target trials and from the remaining pool of whorls on the loop target trials. The position of the target in each array was also randomized.

#### Faces

The stimuli for the face search task were 200 photographs of 200 individuals (100 female and 100 male), sourced from the Face Recognition Grand Challenge database [40]. The photographs selected were full-frontal, eye-aligned images matched for information such as illumination and expression. We converted these photographs to greyscale, cropped them to 180×180 pixels, inverted them, and applied an oval mask to each, removing hair as an obvious gender cue [36].

Forty arrays (20 with a female target singleton among male distractors and 20 with a male target singleton among female distractors) each consisting of 40 photographs, were generated for each participant (see Fig 1A for an example of an array with a female target singleton). We used the same method of sampling targets and distractors in each array as in the fingerprint search task, substituting loop and whorl impressions with inverted female and inverted male faces.

### Procedure

Participants first read an information sheet about the experiment and then completed the two search tasks, one after the other. A random half of our expert and novice participants completed the fingerprint task first, with the remaining half completing the face task first. For the fingerprint task, participants watched an instructional video and were shown examples of a loop target array and a whorl target array. Participants were then presented with 40 arrays of 40 fingerprints one at a time on the computer screen, and were instructed to click on the whorl or loop target as quickly and as accurately as possible. Participants also watched an instructional video about the inverted face task before viewing 40 arrays of 40 inverted faces. In the face task, participants were instructed to click on the inverted female or inverted male target as quickly and as accurately as possible in both tasks.

### Results

For each participant, and each task, we calculated the mean percentage of trials where the target was correctly identified (i.e., clicking on the target image was recorded as a correct response and clicking on a distractor image was recorded as an incorrect response). We also calculated each participant’s average response time over the 40 trials. See Fig 1, for novices’ and experts’ mean percentage of correct responses and mean response times on the fingerprint (Fig 1C) and face task (Fig 1D).

#### Accuracy

Experts outperformed novices at correctly selecting the print and face targets. On average, novices correctly selected the target print 32.19% of the time compared to 92.03% for experts, and novices correctly selected the target face 60.94% of the time compared to 73.59% for experts. We subjected the mean percentage of correct responses to a 2 (Expertise: experts, novices) × 2 (Stimulus Type: fingerprints, faces) mixed analysis of variance. The results revealed a significant main effect of Expertise, *F*(1, 30) = 51.99, *MSE* = .04, *p* < .001, *η*^*2*^G = 0.54 (see [41] on Generalised Eta-Squared as a measure of effect size), but not for Stimulus Type, *F*(1, 30) = 2.15, *MSE* = .02, *p* = .153. The interaction between Expertise and Stimulus Type was also significant, *F*(1, 30) = 45.06, *MSE* = .02, *p* < .001, *η*^*2*^G = 0.33. To clarify this interaction, we performed Bonferroni adjusted pairwise comparisons, comparing the mean percent correct for experts versus novices on the print and inverted face tasks separately. These analyses showed that experts were significantly more accurate than novices at selecting the print targets, *t*(30) = 11.05, *p* < .001, *d* = 4.03, but not the face targets, *t*(30) = 1.87, *p* = .075, *d* = .70. Further analyses also revealed that novices were significantly more accurate at selecting the inverted face targets compared to fingerprints, *t*(15) = 5.22, *p* < .001, *d* = 1.33, but experts were significantly more accurate at selecting the print targets compared to inverted faces, *t*(15) = 4.73, *p* < .001, *d* = 1.87.

#### Speed

The mean response time for experts (11.42 seconds) and novices (11.31 seconds) was similar on the fingerprint task. On the inverted face task, however, novices were much faster (11.12 seconds) than experts (21.65 seconds). We performed a second 2 (Expertise: experts, novices) × 2 (Stimulus Type: fingerprints, faces) mixed analysis of variance on the response time data. We found a significant main effect of Expertise, *F*(1, 30) = 19.34, *MSE* = 23.40, *p* < .001, *η*^*2*^G = 0.33, and for Stimulus Type, *F*(1, 30) = 56.51, *MSE* = 7.14, *p* < .001, *η*^*2*^G = 0.31, but we also found a significant interaction between Expertise and Stimulus Type, *F*(1, 30) = 60.80, *MSE* = 7.14, *p* < .001, *η*^*2*^G = 0.32. Bonferroni corrected pairwise comparisons showed that novices were significantly faster than experts to respond on the inverted face arrays, *t*(30) = 6.69, *p* < .001, *d* = 2.44, but not the fingerprint arrays, *t*(30) = 0.09, *p* = .926, *d* = .03. While novices showed no discernible difference in their response times across the two tasks, *t*(15) = .23, *p* = .926, experts were significantly faster at selecting fingerprints compared to inverted faces, *t*(15) = 9.77, *p* < .001, *d* = 2.59.

#### Correlations

A look at the correlations between the percent correct and response time data for experts compared to novices helps to clarify some of these results. Even though novices were just as fast as experts at selecting fingerprints, their speed was positively correlated with their accuracy, *r*(14) = .51, *p* = .045. No such relationship emerged for novices with inverted faces, although the correlation was of a similar magnitude and in the same direction, *r*(14) = .49, *p* = .054. For experts, the relationship between response times and percentage of correct responses was close to zero in both the fingerprint, *r*(14) = .01, *p* = .977, and inverted face versions of the task, *r*(14) = *-*.17, *p* = .537. Participants’ accuracy on the two tasks was also significantly and positively correlated, *r*(30) = .44, *p* = .012. That is, those who were more accurate with fingerprints tended to be more accurate with inverted faces overall. While this relationship was not reliable within novices or experts, novices’ performance on the two tasks was more highly correlated, *r*(14) = .41, *p* = .0115, than experts’, *r*(14) = .05, *p* = .854.

### Discussion

Without any experience with fingerprints whatsoever, locating fingerprint outliers was more difficult for novices than locating inverted face outliers. Yet, fingerprint experts were able to overcome this asymmetry, showing an advantage in speed and accuracy for locating fingerprint outliers compared to inverted face outliers. Experts were also significantly more accurate with fingerprints compared to novices, despite showing no reliable advantage for inverted faces—a class of stimuli with which they have limited experience. We also found that experts were much slower with the inverted faces than novices, even though their response times were comparable with fingerprints. Taken together, these data suggest that expert examiners are recruiting different processes (or are using the same process much more efficiently) when viewing fingerprints compared to inverted faces—as marked by a boost in accuracy and speed on the task. The significant expert-novice differences for fingerprints and not faces, also provides evidence that fingerprint expertise is domain-specific. This result is consistent with an instance-based account, where expertise is constrained by the similarity of the stimulus to prior experiences.

It is possible that novices were prioritizing speed over accuracy and that slowing them down might dilute the expert accuracy advantage we observed for fingerprints [42]. There was evidence of a speed-accuracy relationship for novices: the faster they responded, the less accurate they were. At the same time, experts’ accuracy was completely independent of their speed on both tasks, and encouraging novices to focus on accuracy across the board is likely to reveal an expert speed advantage for fingerprints. Most telling is the absence of an expertise effect for inverted faces. Novices’ apparent preference for speed over accuracy sets a low bar for experts on both versions of the task, yet experts still displayed no significant advantage for inverted faces. Nevertheless, experts’ higher accuracy with inverted faces, although not significant, warrants further investigation.

In Experiment 2, we further probe the generalizability of fingerprint expertise when matching images that are presented briefly on the screen. Fingerprint experts and undergraduate novices view pairs of prints and inverted faces for 400 milliseconds, then judge whether the prints belong to the same finger or different fingers, and whether the faces depict the same identity or different identities. Keeping exposure time constant restricts the use of speed-accuracy strategies, and the forced choice design allows us to isolate discriminability and response bias (i.e., a tendency to choose one response over another). Presenting two images side-by-side also closely resembles the task that fingerprint examiners encounter day to day, while forcing a decision on a deadline encourages fast, non-analytic or gist processing of the images as a whole [43]. This task structure is vastly different from the visual search task in Experiment 1, where deliberative processing was encouraged by switching target and distractor categories [39]. If fingerprint examiners maintain an accuracy advantage for prints, but not inverted faces, with just a 400 millisecond presentation, then this result would provide further evidence that they are relying on their memory for prints, and not a general visual skill.

## Experiment 2

### Participants and stimuli

#### Participants

In Experiment 2, we tested a second group of 16 practicing fingerprint experts (*Mean Age* = 40.31, *Standard Deviation =* 8.42; 11 female) from four police organizations in Australia (Queensland, The Australian Federal, New South Wales, and Victoria Police) with an average of 11.18 years of experience. Sixteen undergraduates (*Mean Age* = 19.56, *Standard Deviation =* 2.66; 11 female) from The University of Queensland also participated for course credit as a novice comparison group.

#### Stimuli

The fingerprints were the same set used in Experiment 1, except we did not apply a circular mask. One-hundred target fingerprints were randomly paired with either a matching impression recorded on a separate occasion, or a mismatching impression from a random other individual in the set. The images were cropped to 675×675 pixels with the fingerprints isolated in the centre of the frame. The faces were also the same as those used in Experiment 1 without the ovoid mask, so their hair was visible. The photographs were converted to greyscale, cropped to 675×900 pixels, and inverted. Similar to the fingerprints, 100 target identities were randomly paired with either a photograph of the same individual taken on a separate occasion, or a photograph of a different individual. Half of the face pairs were two male faces and the other half, two female faces. No image was repeated in either task.

### Procedure

Each participant read an information sheet about the study and watched an instructional video before completing the fingerprint and inverted face discrimination tasks. As in Experiment 1, we counterbalanced the order in which participants completed the two tasks. For the fingerprint task, experts and novices viewed 100 pairs of fingerprints (50 matching and 50 mismatching) for 400 milliseconds—just enough time for two voluntary eye movements—followed by a 50 millisecond visual mask, and then judged whether fingerprints were left by the same finger or two different fingers. After making a judgment in each case, there was a three-second interval where three short tones were presented to count participants into the next trial. The same procedure was followed for the face discrimination task, except participants judged 100 inverted face pairs (50 matching and 50 mismatching). See Fig 2 for an illustration of a matching trial on the fingerprint discrimination task (Fig 2A), and inverted face discrimination task (Fig 2B).

In both tasks, participants indicated their judgments on the same 12 point, forced-choice confidence rating scale [18], [44]. This scale ranged from 1 (*sure different*) to 12 (*sure same*), where ratings of 1 to 6 indicated a “no match” decision and ratings 7 to 12 indicated a “match” decision. The confidence rating scale gives participants scope to temper their judgments based on their level of confidence while still forcing a binary decision. Confidence levels can also be computed separately using this scale [44]. The image masks were scrambled versions of the preceding pair of images (see Fig 2A for an example), and the order of matching and mismatching trials was randomized for each participant.

### Results

We computed the average discrimination ability (*A'* and response bias (*B''D*) for novices and experts on the fingerprint and inverted face discrimination tasks [45]. These measures allowed us to isolate differences in discrimination ability between novice and experts, independent of their response threshold. See Fig 2, for mean *A'* and *B''D* scores on the fingerprint (Fig 2C) and inverted face task (Fig 2D).

The inverted face identities (*Mean A'* = .58) were more difficult to distinguish than the fingerprints (*Mean A'* = .75). Similar to Experiment 1, participants’ discrimination ability was also significantly and positively correlated on the two tasks, *r*(30) = .50, *p* = .004, and this relationship was significant within novices, *r*(14) = .51, *p* = .044, or experts, *r*(14) = .52, *p* = .039. We first subjected participants’ *A'* scores to a 2 (Expertise: experts, novices) × 2 (Stimulus Type: fingerprints, faces) mixed analysis of variance. We observed a main effect of Stimulus Type, *F*(1, 30) = 84.85, *MSE* = .01, *p* < .001, *η*^*2*^G = 0.44, but no main effect of Expertise, *F*(1, 30) = 1.75, *MSE* = .02, *p* = .195, and the interaction was not significant, *F*(1, 30) = 1.30, *MSE* = .01, *p* = .264. While no effects of expertise were discernible using an omnibus test, we performed two Bonferroni adjusted comparisons comparing the mean *A'*scores for experts and novices on the fingerprint and inverted face tasks separately. These analyses showed that experts (*Mean A'* = .78) were significantly more accurate than novices (*Mean A'* = .72) at discriminating fingerprints, *t*(30) = 2.4, *p <* .023, *d* = .87, but expert (*Mean A'* = .59) and novice (*Mean A'* = .57) discrimination of inverted faces was comparable, *t*(30) = .433, *p* = .668, *d* = .16.

Participants were also more conservative with inverted faces (*Mean B''D* = .17) than with fingerprints (*Mean B''D* = -.00). A 2 (Expertise: experts, novices) × 2 (Stimulus Type: fingerprints, faces) mixed analysis of variance using participants’ *B''D* scores revealed a significant main effect of Stimulus Type, *F*(1, 30) = 5.11 *MSE* = .09, *p* = .031, *η*^*2*^G = .04, but no main effect of Expertise, *F*(1, 30) = .06, *MSE* = .28, *p* = .810, and no interaction, *F*(1, 30) = .20, *MSE* = .09, *p* = .661. Follow up Bonferroni adjusted comparisons showed no significant difference between novices’ (*Mean B''D* = -.00) and experts’ (*Mean B''D* = -.00) *B''D* scores on the fingerprint task, *t*(30) = .00, *p* = .997, or between novices (*Mean B''D* = .13) and experts (*Mean B''D* = .20) on the inverted face task, *t*(30) = .47, *p* = .642.

### Discussion

The results from Experiment 1 suggest that expertise with fingerprints is constrained by prior experience. We compared fingerprint experts’ accuracy and speed at locating categorical outliers to novices who had no prior experience with fingerprints whatsoever. Experts demonstrated superior performance with fingerprints, but not inverted faces. Our novices were possibly responding quickly at the expense of their accuracy, whereas experts displayed no signs of making such a tradeoff. In this second experiment, we compared experts to novices in their ability to discriminate between matching and mismatching fingerprints to their ability to discriminate between matching and mismatching inverted face identities. We presented the images very briefly, preventing the use of idiosyncratic speed-accuracy strategies. Fingerprint experts were more accurate than novices at distinguishing unfamiliar fingerprints, even with just 400 milliseconds to view the images. With the inverted faces, however, experts’ accuracy resembled that of a novice. Consistent with the results of Experiment 1, expertise with fingerprints did not generalize to a new class of stimuli.

## General discussion

We examined whether expertise with distinguishing unfamiliar objects generalizes across tasks and classes of stimuli, in the context of fingerprint identification. We found that fingerprint experts are more accurate than novices at locating fingerprint outliers (i.e., loops and whorls), but not inverted face outliers (i.e., males and females) in a novel visual search task (Experiment 1). Fingerprint experts were also more accurate than novices at distinguishing pairs of fingerprints at a glance, but not so for pairs of inverted faces (Experiment 2). These experts displayed a surprising amount of flexibility in their ability to classify and distinguish fingerprints across two very different tasks, but their expertise seems to have abrupt limits: we found no reliable evidence of generalization to inverted faces at the group level.

These results suggest that expert examiners are bringing some knowledge to bear when viewing fingerprints that novices are not, and that this knowledge is of little use when viewing images that are too far afield. That is, fingerprint experts’ ability to generalize appears to be constrained by their specific set of prior instances [44]. This viewpoint is consistent with exemplar models that emphasize a greater reliance on information stored in memory with expertise [3], [24], [1]. Objects and categories that are similar to previous encounters are identified effortlessly, whereas those that are atypical, unusual, or more distinct from learned dimensions—such as our inverted face images—are more likely to produce novice-like performance [4].

Our data also indicate that a process of comparing information on screen to information stored in memory can operate across tasks. Experts outperformed novices at locating fingerprint outliers in arrays of similar images (Experiment 1), and were also more accurate at distinguishing fingerprints that were presented very briefly (Experiment 2). These findings are at odds with prior work suggesting that experts are poor at adapting to even seemingly trivial changes within their domain [46]. Though, our results can be reconciled with some exemplar models that suggest a process of selectively attending to diagnostic dimensions, depending on the task at hand [47], [48]. It seems that examiners are able to flexibly retrieve information stored in memory in order to navigate completely different levels of specificity [44], and task environments. Further studies are needed to clarify the boundaries of this flexibility and how it develops.

Our point of departure from previous work is in demonstrating that people can develop perceptual expertise even when within-object information is sparse, and that this expertise appears to be domain-specific. Prior work on perceptual expertise has focused on domains where there is an opportunity to accumulate specific experience with the object or category in question. A bird watcher or dog enthusiast can view multiple instances of the same species or breed [9], [49], a car enthusiast can view multiple variants of the same make and model [50], and medical doctors learn more about a particular disease the more times patients present with symptoms of that disease [51]. Forensic examiners don’t have an opportunity to develop a tacit knowledge of what a specific person’s thumb print typically looks like before determining whether another fingerprint belongs to that same, individual thumb. Yet, these experts display expertise with fingerprints that is flexible to distinct changes in the task, but specific to the learned stimulus class.

It is possible that a combination of experience and general perceptual ability is operating; that fingerprint experts make use of a general perceptual skill as well as their memory for fingerprints. At an individual level at least, people’s performance with fingerprints and inverted faces was moderately correlated in each of the experiments, providing some evidence for general perceptual processing across the board. However, this relationship appeared to be driven by novices in Experiment 1, which would suggest a shift from general perceptual processing to more instance-based processing with expertise (although within group correlations were not reliable [24]). We also found no evidence that novices and experts were differentially relying on a general perceptual process in Experiment 2; both novices’ and experts’ discrimination performance with the briefly presented fingerprints and inverted faces was significantly and similarly correlated. Our data, instead, reflect significant differences between novices and experts at the group level that cannot fully be explained by differences in general perceptual processing. Fingerprint experts outperformed novices with fingerprints but not inverted faces on two occasions with two different tasks, which suggests they were making use of general or distributive information stored in memory above and beyond any general perceptual ability. Understanding more about this memory-retrieval process, and the flexibility of expertise with unfamiliar faces and objects, will help to inform general theories of perceptual expertise.

## Supporting information

S1 File Participants’ confidence ratings, hit and false alarm rates, response times and summary data for Experiment 1 (page 1) and Experiment 2 (page 2). (PDF)
