# Supplementary material for: Expertise with unfamiliar objects is flexible to changes in task but not changes in class
Source: PLoS One. 2017 Jun 2;12(6):e0178403. doi: 10.1371/journal.pone.0178403 (PMC5456088; doi:10.1371/journal.pone.0178403)
Supplement: S1 File — (PDF) [file pone.0178403.s001.pdf]

Fingerprints

Fingerprints: Mean percent correct

Subject	Group	A(find loop)	B(find whorl)	PCorrect
1	Novice	0.7	0.8	0.75
2	Novice	0.15	0.4	0.275
3	Novice	0.2	0.25	0.225
4	Novice	0.5	0.55	0.525
5	Novice	0	0.05	0.025
6	Novice	0.25	0.55	0.4
7	Novice	0.65	0.7	0.675
8	Novice	0	0.15	0.075
9	Novice	0.3	0.55	0.425
10	Novice	0.3	0.25	0.275
11	Novice	0.1	0.45	0.275
12	Novice	0.15	0.35	0.25
13	Novice	0.2	0.1	0.15
14	Novice	0.15	0.05	0.1
15	Novice	0.5	0.1	0.3
16	Novice	0.35	0.5	0.425
1	Expert	1	0.9	0.95
2	Expert	1	0.95	0.975
3	Expert	1	1	1
4	Expert	0.95	1	0.975
5	Expert	0.9	0.95	0.925
6	Expert	0.95	0.95	0.95
7	Expert	0.6	1	0.8
8	Expert	0.95	0.95	0.95
9	Expert	0.85	0.9	0.875
10	Expert	1	0.85	0.925
11	Expert	0.95	0.55	0.75
12	Expert	1	1	1
13	Expert	1	0.95	0.975
14	Expert	0.85	0.85	0.85
15	Expert	0.85	0.9	0.875
16	Expert	0.9	1	0.95

Fingerprints: Percent correct summary means

Group	A(find loop)	B(find whorl)	Overall
Novice	28.13%	36.25%	32.19%
Expert	92.19%	91.88%	92.03%

Fingerprints: Mean response time

Subject	Group	A(find loop)	B(find whorl)	RT
1	Novice	16180.05	15022.95	15601.5
2	Novice	10702.05	14927.3	12814.675
3	Novice	7069.2	9019	8044.1
4	Novice	12195.55	13138.75	12667.15
5	Novice	2493.15	2149.65	2321.4
6	Novice	6791.1	10664.45	8727.775
7	Novice	13428.15	14225.15	13826.65
8	Novice	12851.85	9960.9	11406.375
9	Novice	10344.85	20477.2	15411.025
10	Novice	6946.7	10354.7	8650.7
11	Novice	6581.6	7230	6905.8
12	Novice	13386.95	11836.4	12611.675
13	Novice	15703.5	20135.15	17919.325
14	Novice	8670.2	9084.3	8877.25
15	Novice	9858.95	10607.3	10233.125
16	Novice	12708.9	17157.55	14933.225
1	Expert	7938.15	11793.55	9865.85
2	Expert	10950.5	13066.85	12008.675
3	Expert	11103.35	11674.5	11388.925
4	Expert	13523.5	10646.8	12085.15
5	Expert	11422.05	8828.45	10125.25
6	Expert	11785.85	9373.35	10579.6
7	Expert	12064.6	12811.55	12438.075
8	Expert	11875.15	10126.2	11000.675
9	Expert	11897.65	10847.8	11372.725
10	Expert	20526.6	16103.55	18315.075
11	Expert	9297.85	7107.55	8202.7
12	Expert	9721.6	8249.3	8985.45
13	Expert	11307.05	9718.8	10512.925
14	Expert	12964.7	16585.1	14774.9
15	Expert	10224.85	9464.35	9844.6
16	Expert	14844.1	7522.45	11183.275

Fingerprint: Response time summary means

Group	A(find loop)	B(find whorl)	Overall
Novice	10.37	12.25	11.31
Expert	11.97	10.87	11.42

Inverted Faces

Inverted faces: Mean percent correct

Subject	Group	A	B	PCorrect
1	Novice	0.9	1	0.95
2	Novice	0.45	0.5	0.475
3	Novice	0.5	0.65	0.575
4	Novice	0.6	0.75	0.675
5	Novice	0.65	0.65	0.65
6	Novice	0.8	0.9	0.85
7	Novice	0.85	0.5	0.675
8	Novice	0.85	0.5	0.675
9	Novice	0.75	0.25	0.5
10	Novice	0	0.3	0.15
11	Novice	0.7	0.65	0.675
12	Novice	0.55	0.4	0.475
13	Novice	0.85	0.85	0.85
14	Novice	0.15	0	0.075
15	Novice	0.55	0.7	0.625
16	Novice	1	0.75	0.875
1	Expert	0.7	0.9	0.8
2	Expert	0.85	0.75	0.8
3	Expert	0.8	0.9	0.85
4	Expert	0.85	0.8	0.825
5	Expert	0.7	0.85	0.775
6	Expert	0.65	0.45	0.55
7	Expert	0.9	0.75	0.825
8	Expert	0.75	0.6	0.675
9	Expert	0.25	0.75	0.5
10	Expert	0.6	0.65	0.625
11	Expert	0.8	0.8	0.8
12	Expert	0.85	0.95	0.9
13	Expert	0.65	0.9	0.775
14	Expert	0.9	0.85	0.875
15	Expert	0.55	0.7	0.625
16	Expert	0.6	0.55	0.575

Inverted faces: Percent correct summary means

Group	A(find loop)	B(find whorl)	Overall
Novice	63.44%	58.44%	60.94%
Expert	71.25%	75.94%	73.59%

Inverted faces: Mean response time

Subject	Group	A	B	RT
1	Novice	14561.2	17705.6	16133.4
2	Novice	10943.35	25096.95	18020.15
3	Novice	11237.35	11814.3	11525.825
4	Novice	10144.6	7346.7	8745.65
5	Novice	6254.2	9158.4	7706.3
6	Novice	8337.7	10273.85	9305.775
7	Novice	13539.95	15751.1	14645.525
8	Novice	10256.65	11720.15	10988.4
9	Novice	17121.5	9663.1	13392.3
10	Novice	4601.15	5682.4	5141.775
11	Novice	9663.15	12237.35	10950.25
12	Novice	10534.5	9577.55	10056.025
13	Novice	13950.35	10301.4	12125.875
14	Novice	5771.1	7869.55	6820.325
15	Novice	8715.2	8935.35	8825.275
16	Novice	12852.55	14287.1	13569.825
1	Expert	25270	15700.6	20485.3
2	Expert	20745.65	21815.2	21280.425
3	Expert	21179.5	24790.65	22985.075
4	Expert	19029.9	25052.1	22041
5	Expert	22160.8	27437.35	24799.075
6	Expert	23184.65	28570.9	25877.775
7	Expert	14955.6	30121.5	22538.55
8	Expert	15557	21169.8	18363.4
9	Expert	10143.05	19495.95	14819.5
10	Expert	40209.8	36510.4	38360.1
11	Expert	18146.7	21699.65	19923.175
12	Expert	17664.05	16735.6	17199.825
13	Expert	17167.85	22442.35	19805.1
14	Expert	14926	20101	17513.5
15	Expert	19643.05	20077.85	19860.45
16	Expert	19593.5	21493.45	20543.475

Inverted faces: Response time summary means

Group	A	B	Overall
Novice	10.53	11.71	11.12
Expert	19.97	23.33	21.65

[illegible]

[illegible]

[illegible]

[illegible]

[illegible]

[illegible]

[illegible]

[illegible]

[illegible]

[illegible]

[illegible]

[illegible]

Fingerprints

Fingerprints: Corrected Hits and False Alarms

Subject	Group		
1	Novice	Match	No Match
1	Novice	0.63725490196	0.26470588235
2	Novice	0.59803921568	0.24509803921
3	Novice	0.51960784313	0.14705882352
4	Novice	0.65686274509	0.38235294117
5	Novice	0.75490196078	0.32352941176
6	Novice	0.38235294117	0.26470588235
7	Novice	0.69607843137	0.26470588235
8	Novice	0.63725490196	0.32352941176
9	Novice	0.5	0.40196078431
10	Novice	0.67647058823	0.55882352941
11	Novice	0.69607843137	0.57843137254
12	Novice	0.55882352941	0.18627450980
13	Novice	0.83333333333	0.38235294117
14	Novice	0.77450980392	0.40196078431
15	Novice	0.65686274509	0.5
16	Novice	0.73529411764	0.44117647058
1	Expert	0.71568627450	0.36274509803
2	Expert	0.46078431372	0.26470588235
3	Expert	0.77450980392	0.34313725490
4	Expert	0.63725490196	0.26470588235
5	Expert	0.71568627450	0.36274509803
6	Expert	0.06862745098	0.00980392156
7	Expert	0.71568627450	0.16666666666
8	Expert	0.77450980392	0.48039215686
9	Expert	0.75490196078	0.16666666666
10	Expert	0.75490196078	0.34313725490
11	Expert	0.79411764705	0.30392156862
12	Expert	0.83333333333	0.38235294117
13	Expert	0.20588235294	0.04901960784
14	Expert	0.65686274509	0.42156862745
15	Expert	0.83333333333	0.22549019607
16	Expert	0.91176470588	0.48039215686

Fingerprints: A' means

Subject	Group	
1	Novice	A'
1	Novice	0.773
2	Novice	0.764
3	Novice	0.788
4	Novice	0.716
5	Novice	0.802
6	Novice	0.617
7	Novice	0.802
8	Novice	0.739
9	Novice	0.590
10	Novice	0.610
11	Novice	0.612
12	Novice	0.781
13	Novice	0.818
14	Novice	0.776
15	Novice	0.638
16	Novice	0.732
1	Expert	0.762
2	Expert	0.673
3	Expert	0.803
4	Expert	0.773
5	Expert	0.762
6	Expert	0.729
7	Expert	0.856
8	Expert	0.736
9	Expert	0.871
10	Expert	0.793
11	Expert	0.830
12	Expert	0.818
13	Expert	0.732
14	Expert	0.691
15	Expert	0.879
16	Expert	0.826

Fingerprints: B"D means

Subject	Group	
1	Novice	B"D
1	Novice	0.225
2	Novice	0.349
3	Novice	0.686
4	Novice	-0.085
5	Novice	-0.191
6	Novice	0.636
7	Novice	0.096
8	Novice	0.087
9	Novice	0.196
10	Novice	-0.452
11	Novice	-0.517
12	Novice	0.550
13	Novice	-0.512
14	Novice	-0.396
15	Novice	-0.314
16	Novice	-0.374
1	Expert	-0.178
2	Expert	0.529
3	Expert	-0.284
4	Expert	0.225
5	Expert	-0.178
6	Expert	0.999
7	Expert	0.330
8	Expert	-0.521
9	Expert	0.238
10	Expert	-0.233
11	Expert	-0.255
12	Expert	-0.512
13	Expert	0.974
14	Expert	-0.165
15	Expert	-0.186
16	Expert	-0.810

Fingerprints: Summary A'

	A'
Expert	0.78
Novice	0.72

Fingerprints: Summary B"D

	B"D
Expert	-0.00
Novice	-0.00

Inverted Faces

Inverted faces: Corrected Hits and False Alarms

Subject	Group		
1	Novice	Match	No Match
1	Novice	0.67647058823	0.5
2	Novice	0.44117647058	0.20588235294
3	Novice	0.51960784313	0.42156862745
4	Novice	0.48039215686	0.36274509803
5	Novice	0.63725490196	0.48039215686
6	Novice	0.38235294117	0.44117647058
7	Novice	0.57843137254	0.34313725490
8	Novice	0.51960784313	0.44117647058
9	Novice	0.36274509803	0.53921568627
10	Novice	0.63725490196	0.51960784313
11	Novice	0.63725490196	0.63725490196
12	Novice	0.34313725490	0.40196078431
13	Novice	0.51960784313	0.18627450980
14	Novice	0.30392156862	0.44117647058
15	Novice	0.51960784313	0.46078431372
16	Novice	0.57843137254	0.38235294117
1	Expert	0.46078431372	0.44117647058
2	Expert	0.46078431372	0.46078431372
3	Expert	0.81372549019	0.55882352941
4	Expert	0.30392156862	0.08823529411
5	Expert	0.42156862745	0.59803921568
6	Expert	0.14705882352	0.22549019607
7	Expert	0.40196078431	0.12745098039
8	Expert	0.51960784313	0.42156862745
9	Expert	0.48039215686	0.30392156862
10	Expert	0.57843137254	0.42156862745
11	Expert	0.55882352941	0.5
12	Expert	0.63725490196	0.36274509803
13	Expert	0.40196078431	0.40196078431
14	Expert	0.48039215686	0.42156862745
15	Expert	0.46078431372	0.40196078431
16	Expert	0.71568627450	0.44117647058

Inverted faces: A' means

Subject	Group	
1	Novice	A'
1	Novice	0.653
2	Novice	0.707
3	Novice	0.590
4	Novice	0.607
5	Novice	0.637
6	Novice	0.443
7	Novice	0.691
8	Novice	0.573
9	Novice	0.349
10	Novice	0.607
11	Novice	0.500
12	Novice	0.441
13	Novice	0.763
14	Novice	0.373
15	Novice	0.556
16	Novice	0.664
1	Expert	0.519
2	Expert	0.500
3	Expert	0.723
4	Expert	0.737
5	Expert	0.350
6	Expert	0.390
7	Expert	0.749
8	Expert	0.590
9	Expert	0.655
10	Expert	0.636
11	Expert	0.556
12	Expert	0.715
13	Expert	0.500
14	Expert	0.556
15	Expert	0.557
16	Expert	0.719

Inverted faces: B"D means

Subject	Group	
1	Novice	B"D
1	Novice	-0.353
2	Novice	0.660
3	Novice	0.118
4	Novice	0.310
5	Novice	-0.238
6	Novice	0.343
7	Novice	0.165
8	Novice	0.079
9	Novice	0.200
10	Novice	-0.310
11	Novice	-0.511
12	Novice	0.480
13	Novice	0.603
14	Novice	0.487
15	Novice	0.039
16	Novice	0.081
1	Expert	0.194
2	Expert	0.156
3	Expert	-0.694
4	Expert	0.919
5	Expert	-0.040
6	Expert	0.904
7	Expert	0.821
8	Expert	0.118
9	Expert	0.425
10	Expert	-0.000
11	Expert	-0.118
12	Expert	0.000
13	Expert	0.378
14	Expert	0.195
15	Expert	0.270
16	Expert	-0.330

Inverted faces: Summary A'

	A'
Expert	0.59
Novice	0.57

Inverted faces: Summary B"D

	B"D
Expert	0.20
Novice	0.13

[illegible]

[illegible]

[illegible]

[illegible]
